# The Impact of the Entomopathogenic Fungus *Conidiobolus coronatus* on the Free Fatty Acid Profile of the Flesh Fly *Sarcophaga argyrostoma*

**DOI:** 10.3390/insects12110970

**Published:** 2021-10-27

**Authors:** Agata Kaczmarek, Mieczysława Irena Boguś

**Affiliations:** 1 Witold Stefański Institute of Parasitology, Polish Academy of Sciences, Twarda 51/55, 00-818 Warsaw, Poland; slawka@twarda.pan.pl; 2 BIOMIBO, Strzygłowska 15, 04-872 Warsaw, Poland

**Keywords:** cuticle, entomopathogens, free fatty acids, fungal infection, polyunsaturated fatty acids

## Abstract

**Simple Summary:**

The interaction between insect and fungus is characterised on the one hand by the parasite developing more effective strategies of host exploitation, and on the other, by the host mounting increasingly robust defences though Red Queen dynamics or coevolutionary arms races. Furthermore, depending on gene flow and differences in selection pressure between sites, both host and parasite may demonstrate local adaptation to their counterpart or develop more general resistance or offensive traits. As the cuticle is considered the first line of defence of the insect, changes in the FFA profile may well influence susceptibility or resistance to fungal invasion. Our findings indicate that *Sarcophaga argyrostoma* demonstrates stage-specific resistance to *Conidiobolus coronatus* infection and suggests that FFAs play a role in resistance to fungal infection in flesh flies. These findings not only increase our knowledge of the entomopatogenic potential of fungi, but also of the growing level of infection by *C. coronatus* in humans and other mammals. Also, the presented research suggests that FFAs demonstrate antifungal activity which may be helpful in designing new antifungal treatments.

**Abstract:**

The chemical composition of the insect cuticle varies remarkably between species and their life stages. It can affect host resistance and substrate utilization by invading entomopathogen fungi, such as the soil fungus *Conidiobolus coronatus*. In this study, *Sarcophaga argyrostoma* flies were exposed to sporulating *C. coronatus* colonies for 24 h; the pupae were resistant, but the adults demonstrated 60% mortality. Although the pupae demonstrated no sign of infection nor any abnormal development, our findings indicate that after 24 h of contact with the fungus, the pupae demonstrated a 25.2-fold increase in total cuticular free fatty acids (FFAs) and a 1.9-fold decrease in total internal FFAs. Also, the cuticular FFA increased from 26 to 30, while the internal FFA class increased from 13 to 23. In exposed adults, the total mass of cuticular FFAs increased 1.7-fold, while the number of FFAs stayed the same (32 FFAs). Also, the internal FFA class increased from 26 to 35 and the total FFA mass increased 1.1-fold. These considerable differences between adults and pupae associated with *C. coronatus* exposure indicate developmental changes in the mechanisms governing lipid metabolism and spatial distribution in the organism, and suggest that cuticular lipids play a vital role in the defence against pathogenic fungi.

## 1. Introduction

Sarcophagidae is a large family of insects, which are widespread throughout the temperate zone [1,2]. Its members employ a range of feeding strategies, including sarcophagy, coprophagy, and necrophagy. As such, they are known as obligatory and facultative parasitoids, predators, and as myiasis-causing factors [3,4,5,6,7,8,9,10,11,12]. Furthermore, being synanthropic, several Sarcophagidae species may facilitate the mechanical transmission of pathogens to both food and humans; this can have potential consequences for public health [13,14,15]. 

Sarcophagidae, therefore, have a strong negative impact on human and livestock health; as such, there is a pressing need to find safe and effective methods of decreasing their population. Although chemical pesticides are among the most popular methods of controlling insect populations, their disadvantages have spurred the search for new strategies, including the use of entomopathogens [16,17,18,19,20,21], which are natural regulatory factors of insect populations [22]. They have been proposed as eco-friendly alternatives to chemical insecticides, and model organisms for studying insect infection [23,24,25].

As adult flies carry numerous highly pathogenic microorganisms, and can spread them over large areas, they present a serious threat to the health and sanitary safety of humans and farm animals. Although the larvae are more mobile than the pupae, their range of influence is negligible and is limited only to small feeding sites located in carcass, human and animal excrement and biological waste. Pupae generally live in soil, which places them in contact with various microorganisms, including entomopathogenic fungi. This forces an abrupt remodelling of the insect’s organism, leading to the formation of an adult fly. It is, therefore, extremely important to understand the mechanisms that protect these insects against pathogenic microorganisms.

Entomopathogenic fungi are, apart from nematodes, the only insect pathogens able to infect their host by adhering to the surface of the cuticle and penetrating it [26,27]. The insect cuticle is a complex, multifunctional skeleton, and the outermost surface, the epicuticle, plays a key role in protecting insects against fungal infection [28,29]. The epicuticle is composed of a mixture of lipids, proteins and phenolic compounds that accelerate or inhibit fungal growth, and their presence partially determines whether an adherent fungus develops. The layer sits on top of a thicker procuticle, consisting mainly of proteins and chitin [27,30], and is, itself, covered by another layer of saturated and unsaturated hydrocarbons, fatty acids, esters, alcohols, sterols, and aldehydes [30]; the profile of these compounds varies between insect species, the integumental region, and the developmental stage of the insect [31,32,33,34,35,36,37,38].

The entomopathogenic fungus adheres to the host by nonspecific hydrophobic and electrostatic interactions between the conidia and the insect cuticle [39]. The growing hyphae then penetrate the cuticle and enter the host body by a combination of mechanical pressure and the production of cuticle-degrading enzymes [27,39]. The success of the infection is dependent on several factors, such as the structure and composition of the cuticle, the presence of antifungal compounds in the exoskeleton, as well as the efficiency of the cellular and humoral defence reactions of the insect after invasion [40]. The cuticle composition itself strongly influences conidia germination, and this variation results in differences in susceptibility between insect species [27].

Cuticular fatty acids are known to have a range of toxic and fungistatic effects on fungal spore germination, depending on the species of insect; some acids have also been found to have stimulatory effects [31,34,37,41,42,43,44,45,46,47]. The literature data indicate that straight-chain saturated fatty acids, such as caprylic and capric acid, have an inhibitory effect on fungal germination [48,49], while linoleic acid treatment demonstrates a stimulatory effect [50]. Palmitoleic acid enhances the mycelial growth of *Erynia variabilis*, but is toxic to the conidia [51], and the toxic effects of palmitoleic acid can be mitigated by the presence of a sufficient concentration of oleic acid [34]. Moreover, the conidia of *E. variabilis*, grown on water agar, produced secondary (replicative) conidia only in the presence of oleic acid [52]. The presence of C16:0, C18:0, C18:1, C18:2, or C18:3 in the culture media of *Conidiobolus coronatus* inhibits fungal growth and reduces conidia production [47]. Hence, it is important to determine the cuticular fatty acid profile to understand the nature of susceptibility to fungal infection.

*Conidiobolus coronatus* is a cosmopolitan soil fungus that selectively attacks a number of insect species [53]. Previous studies of four medically important fly species (*Calliphora vicina*, *Calliphora vomitoria, Lucilia sericata* (all Diptera: Calliphoridae), and *Musca domestica* (Diptera: Muscidae)) have found pupae to be resistant to *C. coronatus* infection, but the imagines to be susceptible. The enzyme cocktail produced by *C. coronatus*, to degrade the proteins, chitin, and lipids serving as the primary cuticular constituents, contains a mixture of proteases, chitinases, and lipases. The effectiveness of this cocktail is influenced by the concentrations of compounds in the cuticle of the tested insects; this indicates that the cuticular lipids could have heterogeneous functions in protecting against mycosis, insofar that some may be used by the fungus as nutrients, while others may be engaged in resistance [37,41,42].

The main aim of this work was to determine the relationships between the susceptibility to fungal infection of medically important adult and pupal flesh flies, *Sarcophaga (Liopygia) argyrostoma* (Robineau-Desvoidy, 1830), and their free fatty acid (FFA) profiles. The testable hypothesis was that exposure to *C. coronatus* could affect the FFA profiles of the pupae and adult flies.

## 2. Materials and Methods

### 2.1. The Fungus Conidiobolus coronatus

*C. coronatus*, isolate number 3491, originally isolated from *Dendrolaelaps spp.*, was received from the collection of Prof. Bałazy (Polish Academy of Sciences, Research Center for Agricultural and Forest Environment, Poznań). The fungal colonies were routinely cultured in 90 mm Petri dishes on Sabouraud agar (SAB) medium. To increase virulence, the medium was enriched with homogenized *G. mellonella* larvae, to a final concentration of 10% wet weight (SAB-GM). The colonies were incubated at 20 °C under a 12 h photoperiod (L:D 12:12) to stimulate sporulation. The fungal colonies used for the experiments were cultured for seven days.

### 2.2. Insects

*S. argyrostoma* were reared at 25 °C with 70% relative humidity and a 15:9-hour photoperiod. The larvae were fed beef, sugar and water ad libitum. The flies formed puparia 14 days after their larvae hatched from eggs, and the adults emerged 14 days later. The species was confirmed by Prof. Krzysztof Szpila from the Chair of Ecology and Biogeography (Nicolaus Copernicus University in Toruń, Poland). Freshly emerged pupae and six-day-old sexually mature adults were used for experiments. The insects used in the study were sixth generation. These methods expand upon those detailed within our previous work [38].

A culture of the wax moth *G. mellonella* was used as a supplement in the fungal cultures. The moths were reared in glass chambers at 30 °C, 70% relative humidity and in constant darkness on a semi-artificial diet [54]. The fully grown larvae were collected before pupation, surface-sterilized and homogenized. The larvae were also used in the virulence tests routinely performed after each fungus transfer [55]. Percentages of mortality ranged from 80 to 95% in the tested populations.

### 2.3. Infection of Insects

*S. argyrostoma* flies (pupae and adults) were exposed for 24 h at 20 °C to fully grown and sporulating *C. coronatus* colonies, around 10 per Petri dish. The controls were exposed for 24 h to sterile SAB-GM medium. After exposure, the insects were divided into the following two groups: One was transferred to new, clean Petri dishes (imagines with appropriate food), and observed for seven days. The other was treated with water and left to dry, to remove fungal conidia from cuticle surface and then frozen after 24 h exposure to *C. coronatus* and kept at −20 °C until FFA composition was tested. The numbers of individuals used for experiments are presented in Table 1. Each test was performed separately.

The virulence of *C. coronatus* colonies was confirmed by testing on *G. mellonella* larvae treated in the same way as the *S. argyrostoma* pupae and adults.

### 2.4. Extraction of Free Fatty Acids (FFAs)

The cuticle and internal lipid components were extracted from the pupae and adults of *S. argyrostoma*. Firstly, the insects were extracted in 20 mL of petroleum ether for five minutes (extract I) and then a second time in 20 mL of dichloromethane for another five minutes (extract II). These two extracts (I and II) contained the cuticular lipids. The use of petroleum ether minimizes the possible extraction of internal lipids, which are mostly FFAs and glycerides [56]. The third extract was obtained by sonification of insects in 20 mL of dichloromethane for one minute. This extract contained the internal lipids. Each extraction was performed only once due to the small number of available insects. The extracts were placed in glass flasks and then evaporated under nitrogen. The masses of insects and the extracts are presented in Table 1. These methods expand upon those detailed within our previous work [37,38,57].

### 2.5. Derivatization Method

One milligram of each sample and 10 µL of internal standard (19-methylarachidic acid; 1 mg/mL; Merck Millipore) were silylated with 100 μL of N,O-Bis(trimethylsilyl) trifluoroacetamide (BSTFA): chlorotrimethylsilane (TMCS) (99:1) (Merck Millipore) mixture for one hour at 100 °C to obtain trimethylsilyl esters (TMS) of FFAs. The TMS values of the fatty acids were then analysed by GC–MS. The GC-MS analysis used 19-methylarachidic acid as an internal standard (IS) because it separates well from all the sample constituents and was not previously present in the insect samples [58]. These methods expand upon those detailed within our previous work [37,38,42,59].

### 2.6. GC–MS Analyses

The samples were separated and analysed by gas chromatography–mass spectrometry (GC-MS) using high-purity solvents (≥95%, Merck Millipore, Burlington, MA, USA). The GC–MS analyses were carried out on a GCMS-QP2010 system with a mass detector (Shimadzu, Kyoto Japan,). As the carrier gas, helium was used at a column head pressure of 65.2 kPa. A DB-5 MS (Zebron, Phenomenex, Torrance, CA, USA) column was used (thickness 0.25 µm, length 30 m, diameter 0.25 µm). The column oven temperature cycle was maintained at 80 °C for 3 min, then ramped up from 80 to 310 °C at 4 °C/min; the final temperature was then held for 10 min. The ion source temperature was 200 °C and the interface temperature was 310 °C. Split mode was used with a split ratio of 10. All compounds were identified based on the fragmentation patterns and mass-to-charge ions of the TMS derivatives given in the NIST 11 library. The mass spectra of the fatty acid trimethylsilyl esters comprised M+ (molecular ion), [M-15]+, and fragment ions at *m*/*z* 117, 129, 132, and 145. The content of the compounds in the analysed samples was calculated from the chromatogram peak areas. Each sample was analysed in triplicate and the results were expressed as means and standard deviation. Response factors of one were assumed for all constituents. These methods expand upon those detailed within our previous work [36,37,38,42,58,59].

### 2.7. Statistics

Principal component analysis (an unsupervised learning method) was used to visualize differences in the data between control and fungus treated in both cuticular and internal FFA fractions from the pupae and imagines. The test was performed using Past 4.05 software [60]. The normality of the data was checked using the Kolmogorov–Smirnov (K-S) test. As all the variables had normal distributions, they were analysed using Student’s t-test (to compare susceptibility) and ANOVA analysis (to compare FFA profile). The significance level was 95% (*p* < 0.05). STATISTICA software (StatSoft Polska, Cracow, Poland) was used for statistical testing.

## 3. Results

### 3.1. Susceptibility of S. argyrostoma to Fungal Infection

The *S. argyrostoma* imagines and pupae demonstrated different susceptibilities to sporulating *C. coronatus* colonies, with the pupae being resistant and the adults being susceptible to *C. coronatus* infection. The results are shown in more detail in Table 2 and Supplementary Table S1 (raw data). The pupae did not demonstrate any signs of infection nor any signs of fungal penetration through the cuticle. In addition, the metamorphosis continued normally. In contrast, 60% mortality was observed in adults after one day following 24 h exposure to *C. coronatus*, resulting from the ingestion of conidia and/or excretions covering the surface of the fungal colonies; *S. argyrostoma* flies are eager to lick all potential food sources. The SAB-GM medium on which the fungus was grown contains the following ingredients, which attract flies and encourage licking: tryptone, yeast extract, agar, glucose, and homogenized *G. mellonella* larvae.

### 3.2. Effectiveness of Extraction Process

Three types of extraction were performed for the pupal and adult material. The cuticular lipids were obtained in the petroleum ether and dichloromethane extracts (extract I and II, respectively), and the internal lipids in the dichloromethane extracts after sonification (extract III). The total masses of the extracts are shown in Table 1.

Exposure to *C. coronatus* caused changes in the masses of the cuticular and internal extracts in both pupae and adults. A 1.2-fold increase in cuticular lipids and a 16.1-fold decrease in internal lipids per insect was observed in the extracts from the fungus-treated pupae, compared to the untreated pupae. Exposure to the fungus resulted in the proportion of cuticular to internal extracts changing from 1:3.08 in the control pupae to 1:0.16 in the treated pupae.

The fractions from the untreated adults also demonstrated a higher mass of internal lipids (0.44 mg per insect) than cuticular lipids (0.25 mg per insect). Similarly to the pupae, the exposure of adults to *C. coronatus* caused a 1.8-fold increase in the mass of the cuticle extracts and a 3.9-fold decrease in the internal extracts per insect. The ratio of the cuticular to internal fractions was 1:1.76 in the control insects, and 1:0.25 after exposure to fungus.

These extracts were further analysed by GC–MS. A comparison of the FFA profiles of the cuticle surface (sum of extracts I and II), and the internal structures of the pupae and imagines, is given in Table 3, Table 4, Table 5 and Table 6; the raw data are appended in Supplementary Table S2.

### 3.3. GC-MS Analyses

**Figure 1:**
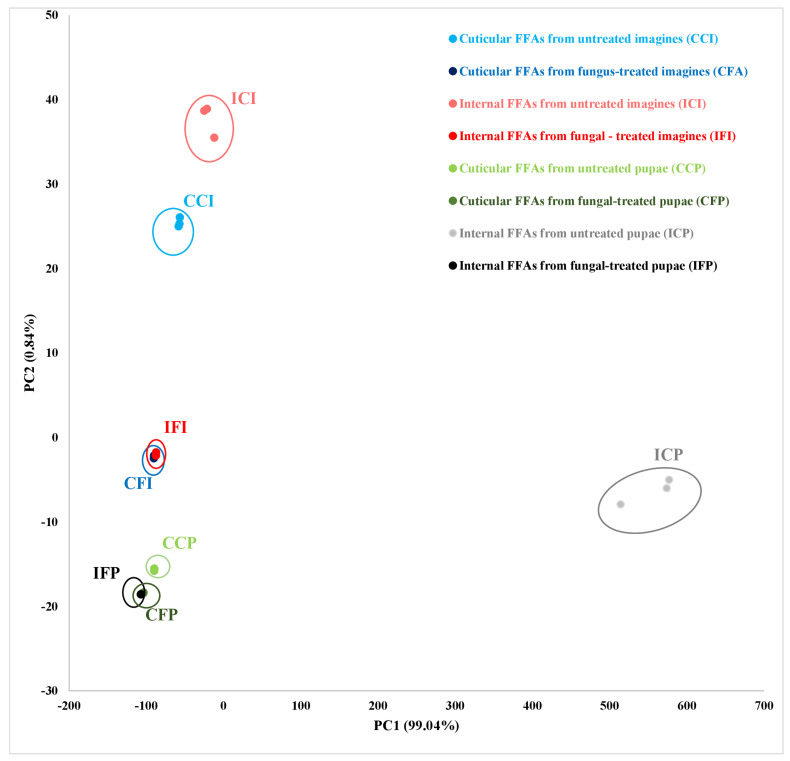
**Principal component analysis (PCA) for *S. argyrostoma* samples from the cuticular and internal FFAs fractions.** FFAs were extracted from control and exposed to *C. coronatus* pupae and adults. The first two principal components cover 99.04% and 0.84% of the variance, respectively, for a total of 99.88% variance.

The principal component analysis (PCA, Figure 1) showed a clear distinction between the concentrations of FFAs in the control group and the group treated with the fungus. The concentrations of FFAs following fungal exposure significantly overlapped in the pupae and imagines; this confirms that the FFA profiles of the cuticular and internal fractions converged after fungal exposure in both developmental stages. The first component explained 99.04% of the variation, and C18:1 and C16:0 represented the largest contribution. The second component explained 0.84% of the variation, with the largest contributions from C16:1.

### 3.4. GC-MS Analyses of Compounds Extracted from Pupae

The concentrations of the individual FFAs extracted from the pupae are presented in Table 3.

The highest total FFA content was observed in the cuticular fraction of the pupae after fungal treatment (1189.69 ± 57.97 μg/g of the insect body); the value was 28.1 times higher than in the controls (47.27 ± 0.26 μg/g of the insect body, F(3,8) = 952.35, *p* < 0.001). However, in the internal fraction, the total FFA content was 1.8 times lower in the extract from the exposed insects (8.53 ± 0.07 μg/g of the insect body) than in the untreated ones (15.78 ± 0.45 μg/g of the insect body).

The FFAs C16:0, C18:1, and C18:0 were found to be dominant in the cuticular and internal extracts from both the untreated and exposed pupae.

The cuticular extracts from the control pupae contained the following 26 FFAs from C6:0 to C32:0: 19 saturated (C6:0–C10:0, C12:0, C14:0-C20:0, C22:0, C24:0, C26:0, C28:0, C30:0, and C32:0) and seven unsaturated (C14:1, C15:1, C16:1, C17:1, C18:2, C18:1, C20:5, and C20:1).

A similar FFA profile was observed in the cuticular extracts from the pupae exposed to fungal infection, with the exception that C20:5 and C20:1 were absent, while C11:0, C15:1, C19:1, C20:4, and C20:3 appeared after exposure to *C. coronatus*. Contact with *C. coronatus* resulted in a significant elevation in the amounts of 19 cuticular FFAs (C6:0, C9:0, C12:0, C14:1, C14:0, C15:0, C16:1, C16:0, C17:1, C17:0, C18:1, C18:0, C19:0, C22:0, C24:0, C26:0, C28:0, and C30:0), ranging from a 643-fold (C14:1, F(3,8) = 501.38, *p* < 0.001) to a 1.8-fold (C18:2, F(3,8) = 23.01, *p* < 0.001) increase. A single FFA (C32:0, F(3,8) = 51.88, *p* < 0.001) demonstrated a two-fold decrease in the exposed pupae (Table 3).

The internal lipids extracted from the control pupae were found to contain 13 FFAs (Table 3) from C6:0 to C18:0, including 10 saturated (C6:0–C10:0, C12:0, C14:0–C16:0, C18:0) and three unsaturated (C16:1, C18:2, C18:1). The total FFA mass was three times lower in the internal extract than in the cuticular fraction. In addition, C14:1, C17:1, C17:0, C19:0, C20:5, C20:1, C20:0, C22:0, C24:0, C26:0, C28:0, C30:0, and C32:0, which were present in the cuticular fractions, were absent from the internal fractions.

After exposure to the fungus, the total mass of internal FFAs in the pupae fell 1.9-fold (*p* < 0.001), and 10 FFAs appeared, which were absent in the untreated pupae (C14:1, C17:1, C20:0, C22:0, C23:0, C24:0, C26:0, C28:0, and C30:0). Exposure also resulted in a decrease in the concentration of 13 FFAs (C6:0, C7:0, C8:0, C9:0, C10:0, C12:0, C14:0, C15:0, C16:1, C16:0, C18:2, C18:1, C18:0), ranging from 23.7-fold (C6:0, F(3,8) = 161.12, *p* < 0.001) to 1.3-fold (C18:1, F(3,8) = 23.01, *p* < 0.001) (Table 3).

The concentrations of glycerol, cholesterol, β-sitosterol, and stigmastanol in the extracts from the pupae are presented in Table 4. Glycerol and cholesterol were observed in all the extracts. Similarly to the FFA content, their levels were elevated 2.7-fold and 2.2-fold in the cuticular fraction following exposure to *C. coronatus*, but decreased 10.2-fold and 14.0-fold (for glycerol F(3,8) = 593.74, *p* < 0.001, and for cholesterol F(3,8) = 614.60, *p* < 0.001) in the internal fraction. Stigmastanol (F(3,8) = 40.71, *p* < 0.001) and β-sitosterol (F(3,8) = 1364.8, *p* < 0.001) were absent in the control pupae, but appeared in both the cuticular and internal fractions after exposure.

**Figure 2:**
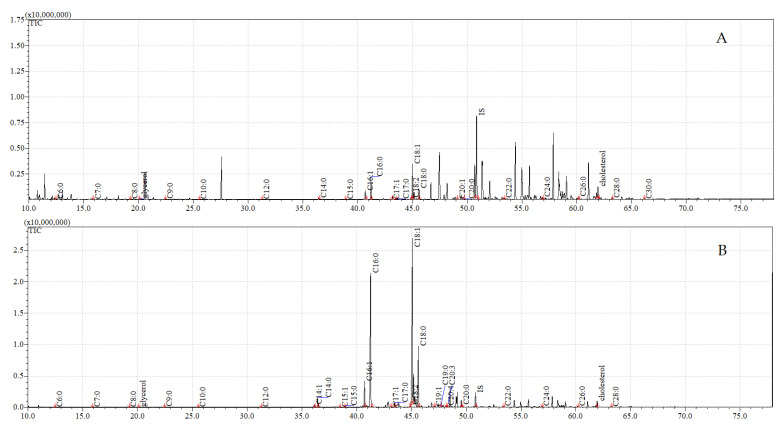
**The total ion current (TIC) chromatogram of fatty acids (TMS esters) of the ether extract (extract I) from control (A) and after *C. coronatus* exposition (B) *S. argyrostoma* pupae**. Internal standard (IS, 19-methylarachidic acid); the following fatty acids and molecular ions: hexanoic acid (C6:0, *m*/*z* = 188), heptanoic acid (C7:0, *m*/*z* = 202), octanoic acid (C8:0, *m*/*z* = 216), nonanoic acid (C9:0, *m*/*z* = 230), decanoic acid (C10:0, *m*/*z* = 244), dodecanoic acid (C12:0, *m*/*z* = 272), tetradecenoic acid (C14:1, *m*/*z* = 298), tetradecanoic acid (C14:0, *m*/*z* = 300), pentadecanoic acid (C15:0, *m*/*z* = 314), hexadecenoic acid (C16:1, *m*/*z* = 326), hexadecanoic acid (C16:0, *m*/*z* = 328), heptadecenoic acid (C17:1, *m*/*z* = 340), heptadecanoic acid (C17:0, *m*/*z* = 342), octadecadienic acid (C18:2, *m*/*z* = 352), octadecenoic acid (C18:1, *m*/*z* = 354), octadecanoic acid (C18:0, *m*/*z* = 356), nonadecanoic acid (C19:0, *m*/*z* = 370), eicosenoic acid (C20:1, *m*/*z* = 382), eicosanoic acid (C20:0, *m*/*z* = 384), docosanoic acid (C22:0, *m*/*z* = 412), etracosanoic acid (C24:0, *m*/*z* = 440), hexacosanoic acid (C26:0, *m*/*z* = 468), octacosanoic acid (C28:0, *m*/*z* = 496), triacontanoic acid (C30:0, *m*/*z* = 524).

**Figure 3:**
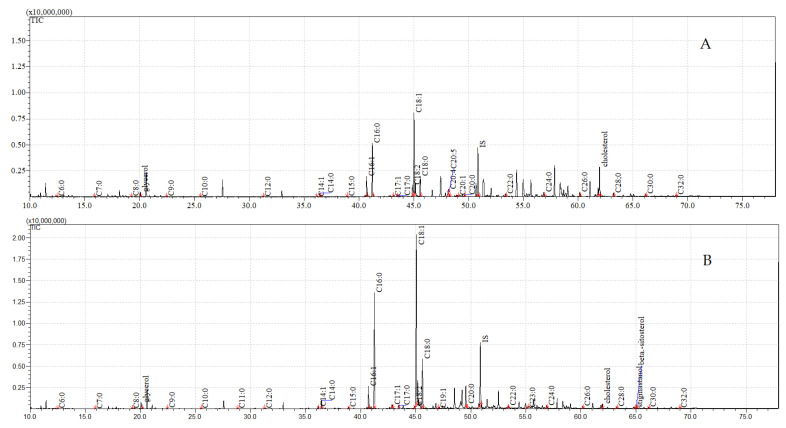
**The total ion current (TIC) chromatogram of fatty acids (TMS esters) of the dichloromethane extract (extract II) from control (A) and after *C. coronatus* exposition (B) *S. argyrostoma* pupae**. Internal standard (IS, 19-methylarachidic acid); the following fatty acids and molecular ions: hexanoic acid (C6:0, *m*/*z* = 188), heptanoic acid (C7:0, *m*/*z* = 202), octanoic acid (C8:0, *m*/*z* = 216), nonanoic acid (C9:0, *m*/*z* = 230), decanoic acid (C10:0, *m*/*z* = 244), undecanoic acid (C11:0, *m*/*z* = 258), dodecanoic acid (C12:0, *m*/*z* = 272), tetradecenoic acid (C14:1, *m*/*z* = 298), tetradecanoic acid (C14:0, *m*/*z* = 300), pentadecenoic acid (C15:1, *m*/*z* = 312), pentadecanoic acid (C15:0, *m*/*z* = 314), hexadecenoic acid (C16:1, *m*/*z* = 326), hexadecanoic acid (C16:0, *m*/*z* = 328), heptadecenoic acid (C17:1, *m*/*z* = 340), heptadecanoic acid (C17:0, *m*/*z* = 342), octadecadienic acid (C18:2, *m*/*z* = 352), octadecenoic acid (C18:1, *m*/*z* = 354), octadecanoic acid (C18:0, *m*/*z* = 356), nonadecenoic acid (C19:1, *m*/*z* = 368), nonadecanoic acid (C19:0, *m*/*z* = 370), eicosatetraenoic acid (C20:4, *m*/*z* = 376), eicosapentaenoic acid (C20:5, *m*/*z* = 374), eicosatrienoic acid (C20:3, *m*/*z* = 378), eicosenoic acid (C20:1, *m*/*z* = 382), eicosanoic acid (C20:0, *m*/*z* = 384), docosanoic acid (C22:0, *m*/*z* = 412), etracosanoic acid (C24:0, *m*/*z* = 440), hexacosanoic acid (C26:0, *m*/*z* = 468), octacosanoic acid (C28:0, *m*/*z* = 496), triacontanoic acid (C30:0, *m*/*z* = 524), dotriacontanoic acid (C32:0, *m*/*z* = 552).

**Figure 4:**
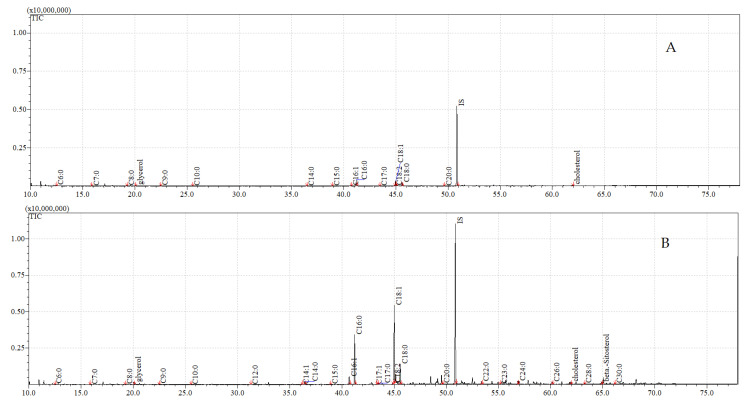
**The total ion current (TIC) chromatogram of fatty acids (TMS esters) of the dichloromethane extract after sonification (extract III) from control (A) and after *C. coronatus* exposition (B) *S. argyrostoma* pupae**. Internal standard (IS, 19-methylarachidic acid); the following fatty acids and molecular ions: hexanoic acid (C6:0, *m*/*z* = 188), heptanoic acid (C7:0, *m*/*z* = 202), octanoic acid (C8:0, *m*/*z* = 216), nonanoic acid (C9:0, *m*/*z* = 230), decanoic acid (C10:0, *m*/*z* = 244), dodecanoic acid (C12:0, *m*/*z* = 272), tetradecenoic acid (C14:1, *m*/*z* = 298), tetradecanoic acid (C14:0, *m*/*z* = 300), pentadecanoic acid (C15:0, *m*/*z* = 314), hexadecenoic acid (C16:1, *m*/*z* = 326), hexadecanoic acid (C16:0, *m*/*z* = 328), heptadecenoic acid (C17:1, *m*/*z* = 340), heptadecanoic acid (C17:0, *m*/*z* = 342), octadecadienic acid (C18:2, *m*/*z* = 352), octadecenoic acid (C18:1, *m*/*z* = 354), octadecanoic acid (C18:0, *m*/*z* = 356), eicosanoic acid (C20:0, *m*/*z* = 384), docosanoic acid (C22:0, *m*/*z* = 412), etracosanoic acid (C24:0, *m*/*z* = 440), hexacosanoic acid (C26:0, *m*/*z* = 468), octacosanoic acid (C28:0, *m*/*z* = 496), triacontanoic acid (C30:0, *m*/*z* = 524).

The total ion current (TIC) chromatograms of the fatty acids (TMS esters) of the ether extract (extract I), dichloromethane extract (extract II), and dichloromethane extract after sonification (extract III), from both the control and exposed pupae, are given in Figure 2, Figure 3 and Figure 4.

### 3.5. GC-MS Analyses of Compounds Extracted from Adults

The untreated adults contained, respectively, 3.0- and 3.4-times higher total masses of FFAs in the cuticular and internal extracts compared to the untreated pupae. In contrast to the pupae, the exposure of adult flies to *C. coronatus* resulted in elevations in the total FFA masses in both the cuticular (1.7-fold) and internal fractions (1.1-fold).

The control adults generally demonstrated a similar cuticular FFA profile to the control pupae; however, the adults lacked C19:0, but included eight FFAs, which were absent from the pupae (C11:0, C12:1, C15;1, C18:3, C19:1, C29:4, C20:3, and C24:1). Of the FFAs that were abundant to both the untreated pupae and untreated adults, C14:0 was 25-fold more plentiful in the adults, C16:1 was 11-fold, C17:1 was eight-fold, C18:2 was six-fold, and C20:5 was 20-fold more plentiful compared to the pupae. In contrast, the following significant differences were found between the internal FFAs of the control adults and the control pupae: thirteen FFAs were present in the adults that were absent in the pupae (C14:1, C17:1, C17:0, C18:3, C20:5, C20:4, C20:0, C22:0, C24:0, C26:0, C28:0, C30:0, and C32:0); of the abundant internal FFAs, C6:0 was 18-fold more abundant in the pupae, C7:0 was seven-fold, C8:0 was 11-fold, and C9:0 was eight-fold more abundant than in the adults, while C16:1 was 23-fold more abundant in the adults, C18:2 was eight-fold, and C18:1 was four-fold more abundant than in the pupae (Table 3 and Table 5).

In the adults, the exposure to *C. coronatus* resulted in the disappearance of C12:1 and C32:0 from the cuticle, and the appearance of C13:0 and C24:1. Exposure was also associated with an increase in the concentration of 20 FFAs (C6:0, C8:0, C9:0, C11:0, C12;0, C14:1, C14:0, C15:1, C15:0, C16:1, C16:0, C17:0, C18:2, C18:1, C18:0, C20:5, C20:4, C20:0, C22:0, and C24:0), ranging from 7.8-fold (C18:0, F(3, 8) = 7.90, *p* = 0.009, MS = 55.31, df = 8.00) to 1.2-fold (C24:0, F(3, 8) = 119.85, *p* < 0.001, MS = 0.001, df = 8.00), as well as a slight decrease in three FFAs (C26:0, C28:0, and C30:0). Exposure also resulted in the appearance of nine internal FFAs (C11:0, C12:1, C13:0, C15:1, C19:1, C19:0, C20:3, C20:1, and C24:1), an increase in the concentration of 18 FFAs (C12:0, C14:1, C14:0, C15:0, C16:0, C17:0, C18:3, C18:2, C18:1, C18:0, C20:5, C20:0, C22:0,C24:0, C26:0,C28:0, C30:0, and C32:0), ranging from seven-fold (C14:1, F(3, 8) = 584.42, *p* < 0.001, MS < 0.001, df = 8.00) to 1.1-fold (C18:1; F(3, 8) = 814.88, *p* < 0.001, MS < 0.001, df = 8.00), and a decrease in five short-chain FFAs (C6:0 to C10:0), ranging from a two-fold decrease to a trace amount (below or equal to 0.001 µg/g body weight; C10:0; F(3, 8) = 42.65, *p* < 0.001, MS < 0.001, df = 8.00). The results are presented in Table 5.

Glycerol and cholesterol were observed in all the extracts (Table 6). β-sitosterol and stigmastanol were found to be absent from the adults, but present in the pupal extracts. The highest concentrations of glycerol and cholesterol were observed in the cuticular fraction after exposure to *C. coronatus*, with these being 1.9-times higher (0.19 µg/g insect body mass) and 1.3 times higher (8.50 µg/g insect body mass) than in the cuticle fraction from the control insects. A higher concentration of cholesterol was also observed in the internal fraction from the imagines exposed to *C. coronatus*; however, no statistically significant differences in glycerol concentration were observed between the internal fractions of the control and exposed adults (0.08–0.07 µg/g insect body mass, respectively).

**Figure 5:**
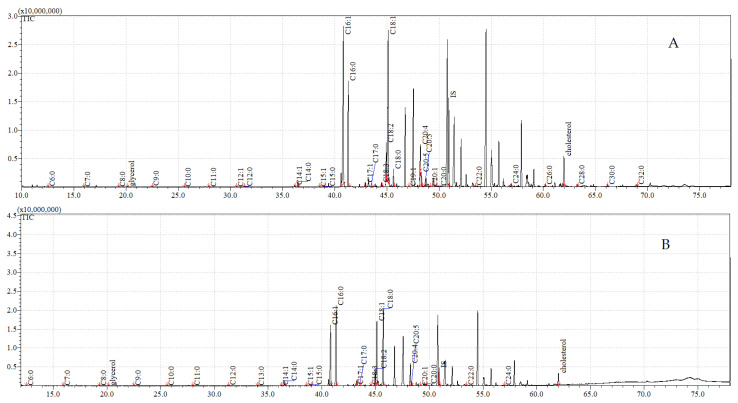
**The total ion current (TIC) chromatogram of fatty acids (TMS esters) of the ether extract (extract I) from control (A) and after *C. coronatus* exposition (B) *S. argyrostoma* imago**. Internal standard (IS, 19-methylarachidic acid); the following fatty acids and molecular ions: hexanoic acid (C6:0, *m*/*z* = 188), heptanoic acid (C7:0, *m*/*z* = 202), octanoic acid (C8:0, *m*/*z* = 216), nonanoic acid (C9:0, *m*/*z* = 230), decanoic acid (C10:0, *m*/*z* = 244), undecanoic acid (C11:0, *m*/*z* = 258), dodecenoic acid (C12:1, *m*/*z* = 270), dodecanoic acid (C12:0, *m*/*z* = 272), tridecanoic acid (C13:0, *m*/*z* = 286), tetradecenoic acid (C14:1, *m*/*z* = 298), tetradecanoic acid (C14:0, *m*/*z* = 300), pentadecenoic acid (C15:1, *m*/*z* = 312), pentadecanoic acid (C15:0, *m*/*z* = 314), hexadecenoic acid (C16:1, *m*/*z* = 326), hexadecanoic acid (C16:0, *m*/*z* = 328), heptadecenoic acid (C17:1, *m*/*z* = 340), heptadecanoic acid (C17:0, *m*/*z* = 342), octadecatrienoic acid (C18:3, *m*/*z* = 350), octadecadienic acid (C18:2, *m*/*z* = 352), octadecenoic acid (C18:1, *m*/*z* = 354), octadecanoic acid (C18:0, *m*/*z* = 356), nonadecenoic acid (C19:1, *m*/*z* = 368), eicosatetraenoic acid (C20:4, *m*/*z* = 376), eicosapentaenoic acid (C20:5, *m*/*z* = 374), eicosatrienoic acid (C20:3, *m*/*z* = 378), eicosenoic acid (C20:1, *m*/*z* = 382), eicosanoic acid (C20:0, *m*/*z* = 384), docosanoic acid (C22:0, *m*/*z* = 412), etracosenoic acid (C24:1, *m*/*z* = 438), etracosanoic acid (C24:0, *m*/*z* = 440), hexacosanoic acid (C26:0, *m*/*z* = 468), octacosanoic acid (C28:0, *m*/*z* = 496), triacontanoic acid (C30:0, *m*/*z* = 524), dotriacontanoic acid (C32:0, *m*/*z* = 552).

**Figure 6:**
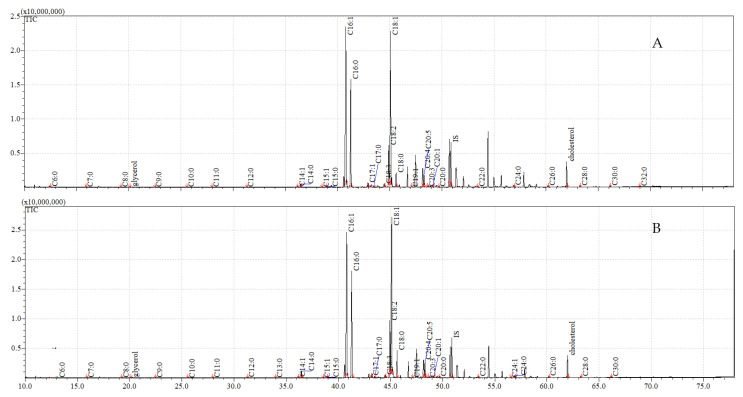
**The total ion current (TIC) chromatogram of fatty acids (TMS esters) of the dichloromethane extract (extract II) from control (A) and after *C. coronatus* exposition (B) *S. argyrostoma* imago**. Internal standard (IS, 19-methylarachidic acid); the following fatty acids and molecular ions: hexanoic acid (C6:0, *m*/*z* = 188), heptanoic acid (C7:0, *m*/*z* = 202), octanoic acid (C8:0, *m*/*z* = 216), nonanoic acid (C9:0, *m*/*z* = 230), decanoic acid (C10:0, *m*/*z* = 244), undecanoic acid (C11:0, *m*/*z* = 258), dodecanoic acid (C12:0, *m*/*z* = 272), tridecanoic acid (C13:0, *m*/*z* = 286), tetradecenoic acid (C14:1, *m*/*z* = 298), tetradecanoic acid (C14:0, *m*/*z* = 300), pentadecenoic acid (C15:1, *m*/*z* = 312), pentadecanoic acid (C15:0, *m*/*z* = 314), hexadecenoic acid (C16:1, *m*/*z* = 326), hexadecanoic acid (C16:0, *m*/*z* = 328), heptadecenoic acid (C17:1, *m*/*z* = 340), heptadecanoic acid (C17:0, *m*/*z* = 342), octadecatrienoic acid (C18:3, *m*/*z* = 350), octadecadienic acid (C18:2, *m*/*z* = 352), octadecenoic acid (C18:1, *m*/*z* = 354), octadecanoic acid (C18:0, *m*/*z* = 356), nonadecenoic acid (C19:1, *m*/*z* = 368), eicosatetraenoic acid (C20:4, *m*/*z* = 376), eicosapentaenoic acid (C20:5, *m*/*z* = 374), eicosatrienoic acid (C20:3, *m*/*z* = 378), eicosenoic acid (C20:1, *m*/*z* = 382), eicosanoic acid (C20:0, *m*/*z* = 384), docosanoic acid (C22:0, *m*/*z* = 412), etracosenoic acid (C24:1, *m*/*z* = 438), etracosanoic acid (C24:0, *m*/*z* = 440), hexacosanoic acid (C26:0, *m*/*z* = 468), octacosanoic acid (C28:0, *m*/*z* = 496), triacontanoic acid (C30:0, *m*/*z* = 524), dotriacontanoic acid (C32:0, *m*/*z* = 552).

**Figure 7:**
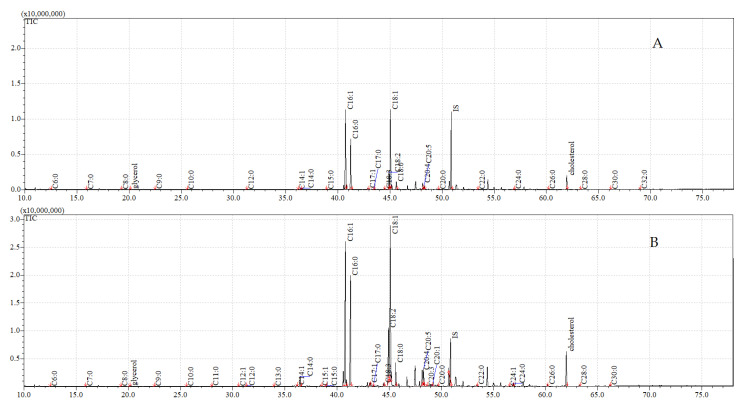
**The total ion current (TIC) chromatogram of fatty acids (TMS esters) of the dichloromethane extract after sonification (extract III) from control (A) and after *C. coronatus* exposition (B) *S. argyrostoma* imago**. Internal standard (IS, 19-methylarachidic acid); the following fatty acids and molecular ions: hexanoic acid (C6:0, *m*/*z* = 188), heptanoic acid (C7:0, *m*/*z* = 202), octanoic acid (C8:0, *m*/*z* = 216), nonanoic acid (C9:0, *m*/*z* = 230), decanoic acid (C10:0, *m*/*z* = 244), undecanoic acid (C11:0, *m*/*z* = 258), dodecenoic acid (C12:1, *m*/*z* = 270), dodecanoic acid (C12:0, *m*/*z* = 272), tridecanoic acid (C13:0, *m*/*z* = 286), tetradecenoic acid (C14:1, *m*/*z* = 298), tetradecanoic acid (C14:0, *m*/*z* = 300), pentadecenoic acid (C15:1, *m*/*z* = 312), pentadecanoic acid (C15:0, *m*/*z* = 314), hexadecenoic acid (C16:1, *m*/*z* = 326), hexadecanoic acid (C16:0, *m*/*z* = 328), heptadecenoic acid (C17:1, *m*/*z* = 340), heptadecanoic acid (C17:0, *m*/*z* = 342), octadecatrienoic acid (C18:3, *m*/*z* = 350), octadecadienic acid (C18:2, *m*/*z* = 352), octadecenoic acid (C18:1, *m*/*z* = 354), octadecanoic acid (C18:0, *m*/*z* = 356), nonadecenoic acid (C19:1, *m*/*z* = 368), nonadecanoic acid (C19:0, *m*/*z* = 370), eicosatetraenoic acid (C20:4, *m*/*z* = 376), eicosapentaenoic acid (C20:5, *m*/*z* = 374), eicosatrienoic acid (C20:3, *m*/*z* = 378), eicosenoic acid (C20:1, *m*/*z* = 382), eicosanoic acid (C20:0, *m*/*z* = 384), docosanoic acid (C22:0, *m*/*z* = 412), etracosenoic acid (C24:1, *m*/*z* = 438), etracosanoic acid (C24:0, *m*/*z* = 440), hexacosanoic acid (C26:0, *m*/*z* = 468), octacosanoic acid (C28:0, *m*/*z* = 496), triacontanoic acid (C30:0, *m*/*z* = 524), dotriacontanoic acid (C32:0, *m*/*z* = 552).

The total ion current (TIC) chromatograms of fatty acids (TMS esters) of the ether extract (extract I), dichloromethane extract (extract II), and dichloromethane extract after sonification (extract III), from control and exposed adults, are given in Figure 5, Figure 6 and Figure 7.

## 4. Discussion

The structure of the host exoskeleton, particularly the composition of lipids present on the cuticle, seems to be a major factor determining the susceptibility or resistance of insect species to *C. coronatus* infection [31,34,35,36,46,61]. Research on *C. vicina* has shown the larvae to be resistant to fungal infection after exposure to sporulating fungal colonies; however, injection of *C. coronatus* conidia resulted in 100% mortality in 24 h. The flies exposed to the fungus did not demonstrate any signs of fungal penetration through the fly cuticle, nor any changes to the internal organs, nor any mobilization of haemocytes to eliminate the fungal pathogen; in contrast, those injected with *C. coronatus* conidia suffered profound damage to the internal organs. Hence, in these Dipteran flies, it appears that a pivotal role in the resistance to fungal infection was played by the cuticle [62]; this is supported by further observations that *C. vicina* larvae have thicker cuticles than those of *G. mellonella* or *D. pini*, which are also more susceptible to fungal infection [62]. The role of cuticular FFAs in the resistance to fungal infection by *C. vicina*, *D. pini*, and *G. mellonella* larvae is also described by Gołębiowski et al. [31].

Our findings indicate that the effects of *C. coronatus* exposure on *S. argyrostoma* vary considerably between developmental stages, with pupae being resistant to infection and adults being susceptible. This result is consistent with previous studies of other flies from the order Diptera, such as *C. vomitoria, C. vicina, L. sericata*, and *M. domestica* [32,34,41,61,62]. While there was no apparent sign of infection on the cuticle of the *S. argyrostoma* imagines, studies have shown a significant mortality rate of adult flies. Assuming that the adult flies were licking the sporulating fungus during the present study, it is likely that the route of infection was by ingestion of fungal spores, which would quickly germinate inside the gut. The growing hyphae release toxic metabolites [42,53,63,64,65], which could promptly kill the flies. In contrast, fungus can only invade the pupae via the cuticle.

Changes in cuticle lipid profiles, occurring during normal development and after contact with the fungus, are the basis for selecting antifungal substances. The fatty acid contents of insects can vary according to their developmental stage, temperature, and dietary regime [38,42,57,59]. A previous study describing the metamorphosis-related changes in the FFA profiles of *S. argyrostoma* indicated the presence of C23:0 and C25:0 only in larvae, C28:0 in the pupal cuticle, and C12:1 and C18:3 in internal extracts from adults. The present research confirms the presence of C28:0 in both the pupae and adults. The occurrence of this FFA is quite unique for insects. It is an aliphatic primary acid, which has been shown to be an antibiofilm and anti-adherence agent against *Streptococcus mutans* (Lactobacillales: Streptococcaceae) [66]; it has, so far, only been detected in the cuticular wax of the honey bee *Apis mellifera* (Hymenoptera: Apidae) [67] and in the cuticular fraction from the larvae and pupae of *Dendrolimus pini* (Lepidoptera: Lasiocampidae) [33]. It is important to note that this FFA is absent in extracts from species considered as significant tools in forensics, such as *C. vicina* [68], *C. vomitoria* [34], and *S. carnaria* [46].

Our present analyses indicate that C12:1, C13:0, C18:3, and C24:1 were present in imagines, but not in pupae. Similarly, previous studies on *S. argyrostoma* found the FFAs C12:1 and C18:3 to only be present in extracts from imagines [37], suggesting that they may be characteristic of this stage of development in this species. In contrast, the FFA C12:1 has been found in extracts from insects that are highly resistant to infection by the entomopathogenic fungus *C. coronatus: Dermestes ater* and *Dermestes maculatus* (Coleoptera; Dermestidae) larvae, pupae, and adults (both female and male) [69], imagines of *C. vomitoria* [34], *Blatella germanica* (Blattodea: Ectobiidae), and *Blatta orientalis* (Blattodea: Blattidae) oothecae [37].

In turn, the FFA C13:0 was observed in the extract from *G. mellonella* imagines [42] and the internal extract from *B. orientalis* oothecae [37], in the larvae and puparia of *C. vicina* [31], and in three development stages (larvae, pupae, and imagines) of *C. vomitoria* [34]. Although this FFA has been found to demonstrate antifungal activity, and to have a negative impact on the growth and sporulation of *C. coronatus* [43,46], Wrońska et al. reported a high positive correlation between the concentration of C13:0 in the cuticle of *G. mellonella* imagines and the effectiveness of the enzyme cocktail produced by *C. coronatus* when breaching the cuticle; this may suggest that it has a positive impact on the fungus [42]. A strong positive corelation was also observed between the concentration of C13:0 in the cuticle of *C. vicina* and the effectiveness of proteases, chitinases, and lipases produced by *C. coronatus* in degrading the main cuticular constituents, viz. the proteins, chitin, and lipids [41].

The FFA C24:1 is an uncommon lipid in insect tissues. In the present study, it was only detected in the fractions from imagines exposed to infection by *C. coronatus*. However, it has previously been observed in extracts from *Sphex flavipennis* (Hymenoptera: Apocrita), mostly in the heads of wasps [70], and in powders obtained from the house cricket *Acheta domesticus* (Orthoptera: Gryllidae) and mealworm *Tenebrio molitor* (Coleoptera: Tenebrionidae) [71]. The FFA C24:1, n-9 Δ15 (cis-15-tetracosenoic acid), or nervonic acid (NA), is an important component in myelin biosynthesis in the central and peripheral nervous system. Myelin is generally localized to the sphingomyelin of animal cell membranes, where it has been proposed to enhance human brain function [72]. Several filamentous fungi and bacteria, such as *Macrophomina phaseolina* (Botryosphaeriales: Botryosphaeriaceae), *Francisella tularensis* (Thiotrichales: Francisellaceae), or *Mortierella capitata* (Mortierellales: Mortierellaceae), are capable of accumulating NA [73], which might suggest that the C24:1 observed in the extracts from adults is produced by *C. coronatus* during fungal exposure (data not presented).

In turn, in the pupal extract, the FFA C23:0 was found to be present. It has also been observed in cuticular extracts from *D. pini* larval exuviae [33], in the whole-body extracts of *Allomyrina dichotoma* (Coleoptera: Scarabaeidae) and *Protaetia brevitarsis* (Coleoptera: Scarabeidae), and the larval extracts of *T. molitor* and *Cirina forda* (Lepidoptera: Saturniidae), as well as in adult *Teleogryllus emma* (Orthoptera: Gryllidae) and *Rhynchophorus palmarum* (Coleoptera: Curculionidae) [74,75,76,77,78]. It has also been observed in male *Hylobius abietis* (Coleoptera: Curculionidae) after *Beauveria bassiana* (Hypocreales: Cordycipitaceae) exposure [79].

Changes in the thickness and composition of the cuticle can increase the resistance of insects by slowing the penetration of a pathogen or insecticide. In addition, transporters of cuticular lipids can be overexpressed in the epidermis, which can improve insecticide resistance by inhibiting insecticide penetration [80].

In the present study, all the pupal and adult extracts demonstrated a gain in mass in the cuticular fraction, following exposure to the fungus, and a loss of mass in the internal extracts. Similarly, the total concentration of all the cuticular FFAs was higher after fungal treatment. In the pupae, which were resistant to the fungal infection, the extract mass increased by only 25 µg per insect; however, the sum of FFAs increased 28 times. This result might suggest that exposure to *C. coronatus* promotes the translocation of FFAs from inside the body to the cuticle of *S. argyrostoma*, and this may serve as a protective mechanism against fungal infection in the pupae. An important finding is that exposure of the pupae to the fungus resulted in an increase in the concentrations of C6:0, C9:0, C12:0, C14:0, C15:0, C16:1, C16:0, C18:1, and C18:0 in the cuticular fraction, accompanied by a decrease in the internal fraction, which might suggest the translocation of these FFAs from the insect body to the cuticle in response to *C. coronatus*. However, more research is needed to confirm this.

After exposure, C11:0, C15:1, C24:0, C23:0, and C23:0 were detected in the cuticular fraction of the pupae, and C17:1, C17:0, and C20:0–C30:0 in the internal fraction. In addition, ß-sitosterol and stigmastanol were observed in both the cuticular and internal fractions. These changes may also be associated with fungal exposure, but, again, more research is needed to confirm this. The lack of C20:5 and C20:1 in the exposed pupae might be due to these acids being used by the fungus as a nutrient; however, this also requires further study.

In the present study, the imagines demonstrated increases in FFA levels in both the cuticular and internal fractions. This change may be the response of the insect to counter fungal infection, as it could protect the insect from both cuticle penetration and the activity of fungal toxins or infection inside the body. In addition, the exposed adults demonstrated the presence of C13:0 and C24:1 in the cuticle, and C11:0, C12:1, C13:0, C19:1, C19:0, C20:3, C20:1, and C24:1 in the internal fraction; as well as this, C12:1 and C32:0 were absent from the cuticle, which suggests that the imagines have a different reaction to *C. coronatus* exposure than the pupae. However, more research is needed to conclusively determine the high susceptibility of adult flies to *C. coronatus* infection.

The observed increase in FFA concentration might be connected with the lipolytic activity of enzymes produced by *C. coronatus*. Previous studies have found that the enzyme cocktail produced by *C. coronatus* demonstrates low efficacy against cuticles extracted from the pupae, thoraces, and wings of adults from four *Diptera* flies, viz. *M. domestica, C. vomitoria, C. vicina*, and *L. sericata*; all of these demonstrate similar resistance to *C. coronatus* infection as *S. argyrostoma* [41]. Histological studies have also found that the conidia of *C. coronatus* did not germinate on the cuticle of *C. vicina* larvae, which are resistant to fungal infection [62]. Hence, we suppose that the detected FFAs are not released in a result of lipolytic activity of fungus from the cuticle of *S. argyrostoma*, a species with similar resistance to fungal infection as Dipteran flies; however, the efficiency of the conidia against internal tissues and organs remains unknown.

At this point, we cannot exclude the possibility that the observed increase in internal FFAs may not only be a result of the insect defence mechanism, but also might be connected with fungal lipolytic activity against internal structures. In the case of the latter, the higher concentrations of the released FFAs would be accompanied with higher glycerol concentrations; lipases liberate free fatty acids and glycerol as a result of hydrolysing lipids at lipid–water interfases, functioning as glycerol ester hydrolases acting on mono-, di, and tri-glycerides [81]. In the present study, the exposure of adults to *C. coronatus* resulted in an increase in FFA concentration, accompanied, in one case, by a decrease in glycerol content; this might suggest that even the digestion of internal lipids is not a very effective process, or that the digested glycerol is quickly transported to other parts of the body. More research is needed to confirm these theses.

Cuticular fatty acids have a profound effect on fungal spore germination, and have different effects depending on the species of invading fungus, being either toxic, fungistatic, or, occasionally, stimulatory. Previous studies have shown that the FFAs C16:0, C16:1, C18:0, and C18:1 inhibit the growth and sporulation of *C. coronatus* [43,47]. The FFAs C18:3, C20:0, and C20:1 have been found to inhibit not only the growth and sporulation of *C. coronatus*, but also the virulence and toxicity of fungal metabolites released into the medium, even at a concentration of 0.0001% [43,47]. In addition, previous studies on insects that were resistant or susceptible to *C. coronatus* infection have identified a correlation between the concentrations of those FFAs (C16:0, C16:1, C18:0, C18:1 C18:3, C20:0, and C20:1) in the cuticle and the effectiveness of the protease, chitinase and lipase enzyme cocktail produced by *C. coronatus*, degrading the main cuticular constituents (proteins, chitin, and lipids) [37,41,42]. On the other hand, C16:0, C18:1, and C18:0 are favoured by another insect fungal pathogen, *B. bassiana*, and research has shown that supplementing the medium with these FFAs results in increased virulence. Moreover, decreased virulence was observed in a mutant with a loss of CYP P450 function, an enzyme known to display terminal hydroxylation activity against fatty acids. *B. bassiana* mutants also demonstrated reduced virulency in percutaneous infections against *G. mellonella* larvae, but displayed no such reduction when the insect cuticle was bypassed in intra-hemocoel injection assays; this suggests that cuticular FFAs may act as susceptibility factors [81,82,83].

In the present study, C16:0, C16:1, C18:0, and C18:1 FFAs are dominant in both the cuticular and internal fractions, and their concentrations increase after exposure to *C. coronatus*. A considerable increase in their concentration in the cuticle was also observed in *C. vicina* larvae and adults after treatment with the *C. coronatus* metabolite dodecanol [84]. Moreover, research comparing the FFA profiles of three species with differing susceptibility to fungal infection confirmed the presence of C16:0 only in *C. vicina*, which is highly resistant to fungal infection [31].

Several differences can be observed between the control and exposed insects, with regard to fatty acid profiles; for example, in the pupae, C11:0, C15:1, and C19:1 are present in the exposed insects, but not in the controls. This absence has also been observed in a previous study, in extracts from untreated pupae of *S. argyrostoma* [37], which might suggest that they are not typical for this developmental stage of this insect, and appear as a response to fungal infection. However, this suggestion needs to be verified. Each of the following FFAs have antifungal activity: C11:0 and C19:1 inhibit the growth of entomopathogenic fungi, such as *Metharhizium anisopliae* (Hypocreales: Clavicipitaceae) or *B. bassiana* [46], and C11:0 has also shown antifungal activity against *Candida albicans* (Saccharomycetales: Saccharomycetaceae), *Myrothecium verrucaria* (Hypocreales: Stachybotryaceae), *Saccharomyces cerevisiae* (Saccharomycetales: Saccharomycetaceae), *Trichoderma viride* (Hypocreales: Hypocreaceae), and *Trichophyton rubrum* (Onygenales: Arthrodermataceae) [46,85]. Studies have also found C11:0 to have antibacterial properties against *Streptococcus faecalis, S. pyogenes* (Lactobacillales: Enterococcaceae), *Staphylococcus aureus* (Bacillales: Staphylococcaceae), *Corynebacterium sp.* (Corynebacteriales: Corynebacteriaceae), *Nocardia asteroids* (Actinomycetales: Nocardiaceae), *Bacullus larvae* (Bacillales: Paenibacillaceae), *Helicobacter pylori* (Campylobacterales: Helicobacteraceae), *B. cereus, Escherichia coli* (Enterobacterales: Enterobacteriaceae), and *Pseudomonas aeruginosa* (Pseudomonadales: Pseudomonadaceae) [86]. The presence of undecanoic acid was also indicated in cuticular extracts from male *B. germanica*, after exposure to chlorpyrifos [87].

*C. coronatus* infection also caused changes in the concentration of glycerol. Similar to the FFAs, its accumulation was observed in the cuticular fraction from *S. argyrostoma* pupae and adults exposed to the fungus. The presence of glycerol in invertebrates prevents them from freezing and protects them from osmotic stress [88,89,90]; the increase in glycerol concentration in non-diapausing larvae of the flesh fly *S. bullata* occurs as a response to several forms of short-term environmental stress, such as low temperature, anoxia, and desiccation [91]. The accumulation of glycerol in the cuticle might be used to reduce water loss through evaporation [34,91]; however, increased glycerol and lipid mobilization, at least in part, is responsible for the increased appressorial turgor pressure of entomopathogenic *Metarhizium* spp. [92,93].

Another important group of compounds present in insect lipids are sterols. Of these, *S. argyrostoma* mostly displayed accumulation of cholesterol after *C. coronatus* exposure. Similar increases in cholesterol concentration have also been observed in the fungus-sensitive pine weevil *H. abietis* after *B. bassiana* infection [79]; however, cholesterol content was found to decrease in fungus-sensitive male *Tettigonia viridissima* (Orthoptera: Tettigoniidae) after *C. coronatus* treatment [94].

Being an important component of cellular membranes [95], and the precursor for many hormones [96,97], and due to its role in regulating genes involved in developmental processes [98], cholesterol is essential for the metabolism of insects. It has also been found to accumulate in membranes at low temperatures [99]. Insects are obligate sterol auxotrophs and must obtain cholesterol or its precursors from their diet. The fact that arthropods expend considerable energy in sequestering cholesterol in their cuticle suggests that it has functional significance; however, its role, if any, remains obscure [98,100].

Following fungal exposure, the presence of β-sitosterol and stigmastanol was observed in *S. argyrostoma* pupae, but not in control pupae or in adults. Insects use β-sitosterol as a substrate for cholesterol synthesis, and it has been found to contribute to the activation of biting and dietary selection in the silkworm *Bombyx mori* (Lepidoptera: Bombycidae). In addition, the inhibition of β-sitosterol metabolism results in growth inhibition in the tobacco hornworm *M. sexta* [101,102,103]. β-sitosterol demonstrates antibacterial and antifungal activity against *Salmonella typhi* (Enterobacterales: Enterobacteriaceae), *Corynebacterium diphtheriae*, *Bacillus subtilis* (Bacillales: Bacillaceae), and *Fusarium spp* (Hypocreales: Nectriaceae), and inhibits spore germination and germ-tube elongation in *Aspergillus niger* (Eurotiales: Trichocomaceae) and *Botryodiplodia theobromae* (Botryosphaeriales: Botryosphaeriaceae) [104,105]. The addition of β-sitosterol to *A. caespitosus* growth medium increases its inhibitory activity against *C. albicans* [106].

Both β-sitosterol and stigmastanol (5,6-dihydro-β-sitosterol) are intermediate substrates in the metabolism of cholesterol in the Mexican bean beetle *Epilachna varivestis* (Coleoptera: Coccinellidae) [107]. This might suggest that the presence of these sterols in pupae is connected with their role as substrates for cholesterol synthesis; however, again, more research is needed to confirm this thesis. Stigmastanol was also observed in the internal extract from *T. molitor*, after treatment with cyfluthrin-containing insecticide [108].

Fungal infection changes not only the composition of the cuticle, but also activates the immune system and induces the production of various immune molecules, including antibacterial proteins. Research has shown the presence of antifungal protein in the hemolymph of *S. peregrina* larvae (AFP). This protein significantly inhibits the growth of *C. albicans*, but has no effect on bacterial growth; its antifungal activity is increased when it forms a complex with the bactericidal protein sarcotoxin IA [109]. Two other important components of the immune system are nitric oxide (NO) and immune-reactive lysozymes. NO is an immune response molecule against bacteria that is mainly biosynthesized in the haemocytes of *S. argyrostoma*, while lysozymes support early immune responses in flesh fly larvae against invading bacteria by degrading bacterial cells and debris [110].

The biosynthesis of NO and lysozymes in *S. argyrostoma* larvae, arising in response to infection, is mediated and coordinated by eicosanoids [110], which are mediators of insect cellular and humoral immunity, and various metabolites of C20:4 (arachidonic acid) [111,112]. They not only mediate this biosynthesis of NO and lysozyme [110], but also participate in the LPS-dependent activation of the IMD pathway in *S. peregrina* [113]. C20:4 has also been found to be present at 90-times higher concentrations in the internal extracts of female *S. carnaria* than males; it has been proposed that the compound may play an important role in vitellogenesis [46]. Clements et al. [114] propose that arachidonic acid may also play a role in the resistance of the Colorado potato beetle *Leptinotarsa decemlineata* (Coleoptera: Chrysomelidae) to neonicotinoid insecticide, and suggest that this may be associated with its regulatory role in cytochrome P450-dependent insecticide detoxification pathways; however, Stanley and Kim postulate that it is evolutionarily advantageous for insects to maintain a low level of polyunsaturated fatty acid (PUFA), to reduce oxidative stress at the intracellular level, due to their high sensitivity to oxygen damage [115].

## 5. Conclusions

In conclusion, the internal and surface FFA compositions of *S. argyrostoma* pupae and adults changed after exposure to *C. coronatus*; most strikingly, the developmental stages demonstrated considerable discrepancy in the reactions to fungus exposure, with the pupae being resistant and the adults being susceptible to infection. As the cuticle is considered the first defence mechanism of the insect, changes in the FFA profile may well influence the susceptibility or resistance of the insects to fungal invasion. In extracts from pupae exposed to fungus, the concentrations of individual FFAs (C6:0–C10:0, C12:0, C14:0, C15:0, C16:1, C16:0, C18:2, C18:1, and C18:0) increased in the cuticular fraction, but decreased in the internal fraction. This might suggest that *C. coronatus* treatment promotes the translocation of these FFAs, and this may be one of the defence mechanisms used against fungal infection. In imagines, the increase in FFAs observed in both the cuticular and internal fractions might reflect the response to fungal infection, not only to protect against cuticle penetration, but also to protect from spore poisoning or infection inside the body. The increase in the FFA C20:4, which has immunomodulatory activities in internal extracts, might also suggest activation of the insects immune system.

## Figures and Tables

**Table 1:** The numbers of *Sarcophaga argyrostoma* pupae and adults used for extraction and masses of extracts obtained.

Treatments:		N	Insects Mass [g]	Extract Mass					
				mg			mg/Insect		
				I	II	III	I	II	III
pupae	control	40	0.83	4.53	1.12	17.37	0.113	0.028	0.434
	exposure to *C. coronatus*	18	0.24	2.08	0.88	0.47	0.116	0.049	0.027
adults	control	57	5.71	5.94	8.36	25.17	0.104	0.147	0.442
	exposure to *C. coronatus*	47	4.78	13.97	7.29	5.25	0.297	0.156	0.112

N—total number of individuals; I—petroleum ether extract; II—dichloromethane extract; III—dichloromethane extract after sonification.

**Table 2:** The susceptibility of *S. argyrostoma* pupae and adults to fungal infection.

Insect Treatment		N	Percent of Flies Hatched from Pupae [%±SD]	Mortality of Imagines [%±SD]
pupae	control	30	80.00 ± 8.00	7.41 ± 12.83 ^#^
	exposure to *C. coronatus*	30	80 ± 0.00	8.33 ± 7.22 ^#^
adults	control	30		3.33 ± 5.77 *
	exposure to *C. coronatus*	30		60.00 ± 10.00 *

N—total number of individuals. SD—standard deviation; ^#^ as 100% was counted the number of flies hatched from pupae; * statistically significant differences between control and exposed to fungal infection insects (Student’s *t*-test, *p* < 0.05). See Supplementary Table S1 for raw data.

**Table 3:** Fatty acid content of the cuticular and internal lipids extracted from the pupae of *Sarcophaga argyrostoma* (μg/g of insect body ± SD).

FFA	Cuticular		Internal	
	Control	Exposure to *C. coronatus*	Control	Exposure to *C. coronatus*
Hexanoic acid C6:0	0.17 ± 0.01 ^A^	0.37 ± 0.02 ^A^	0.71 ± 0.06 ^A^	0.03 ± 0.00 ^A^
Heptanoic acid C7:0	0.02 ± 0.01 ^A^	0.06 ± 0.02 ^B^	0.14 ± 0.02 ^A,B^	0.01 ± 0.00 ^B^
Octanoic acid C8:0	0.26 ± 0.06 ^A^	0.31 ± 0.06 ^B^	0.33 ± 0.01 ^C^	0.02 ± 0.00 ^A,B,C^
Nonanoic acid C9:0	0.28 ± 0.00 ^A^	1.16 ± 0.08 ^A^	0.65 ± 0.04 ^A^	0.04 ± 0.00 ^A^
Decanoic acid C10:0	0.06 ± 0.00	0.19 ± 0.07 ^A,B^	0.06 ± 0.02 ^A^	0.01 ± 0.00 ^B^
Undecanoic acid C11:0	ND ^A^	0.04 ± 0.01 ^A,B,C^	ND ^B^	ND ^C^
Dodecanoic acid C12:0	0.10 ± 0.01 ^A^	0.38 ± 0.05 ^A,B^	0.07 ± 0.02 ^B^	0.01 ± 0.00 ^A^
Tetradecenoic acid C14:1	0.01 ± 0.01 ^A^	6.43 ± 0.41 ^A,B,C^	ND ^B^	0.03 ± 0.00 ^C^
Tetradecanoic acid C14:0	0.85 ± 0.02 ^A^	22.46 ± 1.00 ^A,B,C^	0.23 ± 0.03 ^B^	0.13 ± 0.00 ^C^
Pentadecenoic acid C15:1	ND ^A^	0.10 ± 0.05 ^A,B,C^	ND ^B^	ND ^C^
Pentadecanoic acid C15:0	0.15 ± 0.01 ^A^	4.04 ± 0.21 ^A,B,C^	0.07 ± 0.02 ^B^	0.03 ± 0.00 ^C^
Hexadecenoic acid C16:1	4.29 ± 0.12 ^A^	59.94 ± 3.02 ^A,B,C^	0.76 ± 0.05 ^B^	0.36 ± 0.00 ^C^
Hexadecanoic acid C16:0	11.42 ± 0.12 ^A^	386.27 ± 12.76 ^A,B,C^	5.60 ± 0.18 ^B^	2.41 ± 0.3 ^C^
Heptadecenoic acid C17:1	0.10 ± 0.08 ^A^	2.63 ± 0.22 ^A,B,C^	ND ^B^	0.06 ± 0.00 ^C^
Heptadecanoic acid C17:0	0.21 ± 0.00 ^A^	10.85 ± 0.67 ^A,B,C^	ND ^B^	0.05 ± 0.00 ^C^
Octadecadienoic acid C18:2	1.50 ± 0.07 ^A^	2.73 ± 0.71 ^A,B^	0.39 ± 0.03 ^B^	0.06 ± 0.00 ^A^
Octadecenoic acid C18:1	17.73 ± 0.15 ^A^	517.97 ± 25.16 ^A,B,C^	4.91 ± 0.23 ^B^	3.85 ± 0.06 ^C^
Octadecanoic acid C18:0	5.15 ± 0.11 ^A^	151.16 ± 7.66 ^A,B,C^	3.14 ± 0.09 ^B^	0.93 ± 0.02 ^C^
Nonadecenoic acid C19:1	ND ^A^	1.05 ± 0.27 ^A,B,C^	ND ^B^	ND ^C^
Nonadecanoic acid C19:0	0.01 ± 0.00 ^A^	0.80 ± 0.13 ^A,B,C^	ND ^B^	ND ^C^
Eicosapentaenoic acid C20:5	0.09 ± 0.00 ^A,B,C^	ND ^A^	ND ^B^	ND ^C^
Eicosatetraenoic acid C20:4	ND ^A^	3.04 ± 0.30 ^A,B,C^	ND ^B^	ND ^C^
Eicosatrienoic acid C20:3	ND ^A^	1.03 ± 0.10 ^A,B,C^	ND ^B^	ND ^C^
Eicosenoic acid C20:1	0.16 ± 0.02 ^A,B,C^	ND ^A^	ND ^B^	ND ^C^
Eicosanoic acid C20:0	0.29 ± 0.01 ^A,B^	0.40 ± 0.13 ^C,D^	ND ^A,C^	0.05 ± 0.00 ^B,D^
Docosanoic acid C22:0	0.63 ± 0.04 ^A,B^	1.99 ± 0.24 ^A,B^	ND ^A^	0.11 ± 0.00 ^B^
Tricosanoic acid C23:0	ND^A^	0.10 ± 0.01 ^A,B^	ND ^B^	0.03 ± 0.00 ^A,B^
Tetracosanoic acid C24:0	0.97 ± 0.03 ^A,B^	2.76 ± 0.23 ^A,B^	ND ^A^	0.13 ± 0.00 ^B^
Hexacosanoic acid C26:0	1.20 ± 0.04 ^A,B^	2.57 ± 0.22 ^A,B^	ND ^A^	0.11 ± 0.00 ^B^
Octacosanoic acid C28:0	0.84 ± 0.01 ^A,B^	1.31 ± 0.12 ^A,B^	ND ^A^	0.05 ± 0.00 ^B^
Triacontanoic acid C30:0	0.50 ± 0.05	0.74 ± 0.05 ^A,B^	ND ^A^	0.03 ± 0.00 ^B^
Dotriacontanoic acid C32:0	0.28 ± 0.04 ^A,B^	0.14 ± 0.03 ^A,B^	ND ^A^	ND ^B^
Sum of FFA	47.27 ± 0.26 ^A^	1189.69 ± 57.97 ^A,B,C^	15.87 ± 0.45 ^B^	8.53 ± 0.07 ^C^

FFA—free fatty acid; SD—standard deviation; ND—not detected; statistically significant differences (ANOVA, post hoc Tukey’s HSD) between concentration of FFAs are marked with the same letters (^A,B,C^), see Supplementary Table S2 for raw data.

**Table 4:** Other compounds extracted from *Sarcophaga argyrostoma* pupae (μg/g of insect body ± SD).

	Cuticular		Internal	
	Control	Exposure to *C. coronatus*	Control	Exposure to *C. coronatus*
Glycerol	0.67 ± 0.03 ^A^	1.82 ± 0.08 ^A^	0.92 ± 0.02 ^A^	0.09 ± 0.00 ^A^
Cholesterol	6.51 ± 0.25 ^A,B^	14.48 ± 0.70 ^A,B^	1.26 ± 0.07 ^A^	0.09 ± 0.01 ^B^
β-Sitosterol	ND ^A^	0.24±0.01 ^A,B,^	ND ^B^	0.10 ± 0.01 ^A,B^
Stigmastanol	ND ^A^	0.53±0.11 ^A,B^	ND ^B^	0.32 ± 0.02 ^A,B^

SD—standard deviation; ND—not detected; statistically significant differences (ANOVA, post hoc Tukey’s HSD) between concentration of compounds are marked with the same letters (^A,B^); see Supplementary Table S2 for raw data.

**Table 5:** Fatty acid content of the cuticular and internal lipids extracted from *Sarcophaga argyrostoma* adults (μg/g of insect body ± SD).

FFA	Cuticular		Internal	
	Control	Exposure to *C. coronatus*	Control	Exposure to *C. coronatus*
Hexanoic acid C6:0	0.06 ± 0.00 ^A^	0.09 ± 0.01 ^A^	0.04 ± 0.00 ^A^	0.02 ± 0.00 ^A^
Heptanoic acid C7:0	0.02 ± 0.00 ^B^	0.04 ± 0.01 ^A,B^	0.02 ± 0.00 ^A^	0.01 ± 0.00 ^B^
Octanoic acid C8:0	0.04 ± 0.00 ^B^	0.08 ± 0.01 ^A,B^	0.03 ± 0.00 ^A^	0.02 ± 0.00 ^A,B^
Nonanoic acid C9:0	0.09 ± 0.01 ^B^	0.16 ± 0.01 ^A,B^	0.08 ± 0.01 ^A^	0.04 ± 0.00 ^A,B^
Decanoic acid C10:0	0.02 ± 0.00 ^B^	0.03 ± 0.00 ^A,B^	0.02 ± 0.00 ^A^	trace amount ^A,B^
Undecanoic acid C11:0	0.05 ± 0.01 ^A^	0.16 ± 0.01 ^A,B^	ND ^A,B^	0.05 ± 0.00 ^B^
Dodecenoic acid C12:1	0.01 ± 0.00 ^A,B^	ND ^B^	ND ^A^	0.01 ± 0.00 ^A,B^
Dodecanoic acid C12:0	0.09 ± 0.00 ^A^	0.34 ± 0.01 ^A^	0.04 ± 0.01 ^A^	0.07 ± 0.00 ^A^
Tridecanoic acid C13:0	ND ^A^	0.18 ± 0.02 ^A,B^	ND ^B^	0.03 ± 0.00 ^A,B^
Tetradecenoic acid C14:1	0.25 ± 0.01 ^A^	0.42 ± 0.01 ^A^	0.07 ± 0.01 ^A^	0.10 ± 0.01 ^A^
Tetradecanoic acid C14:0	0.82 ± 0.00 ^A^	2.78 ± 0.14 ^A,B^	0.33 ± 0.01 ^A,B^	0.97 ± 0.00 ^B^
Pentadecenoic acid C15:1	0.02 ± 0.00 ^A,B^	0.06 ± 0.01 ^A,B^	ND ^A^	trace amount ^B^
Pentadecanoic acid C15:0	0.10 ± 0.00 ^A^	0.39 ± 0.07 ^A,B,C^	0.05 ± 0.01 ^B^	0.08 ± 0.00 ^C^
Hexadecenoic acid C16:1	46.61 ± 0.46 ^A,B^	58.90 ± 1.13 ^A,B^	17.53 ± 0.19	17.24 ± 0.19
Hexadecanoic acid C16:0	25.60 ± 0.36 ^A,B^	52.12 ± 8.16 ^A,B^	10.91 ± 0.14 ^A^	12.11 ± 0.12 ^B^
Heptadecenoic acid C17:1	0.80 ± 0.20 ^A,C^	0.80 ± 0.12 ^B,D^	0.21 ± 0.01 ^A,B^	0.18 ± 0.03 ^C,D^
Heptadecanoic acid C17:0	0.13 ± 0.01 ^A^	0.34 ± 0.08 ^A,B,C^	0.05 ± 0.01 ^B^	0.07 ± 0.00 ^C^
Octadecatrienoic acid C18:3	0.76 ± 0.01 ^A,C^	0.79 ± 0.02 ^B,D^	0.23 ± 0.01 ^A,B^	0.28 ± 0.00 ^C,D^
Octadecadienoic acid C18:2	8.93 ± 0.18 ^A^	16.74 ± 0.33 ^A^	3.47 ± 0.06 ^A^	5.75 ± 0.12 ^A^
Octadecenoic acid C18:1	45.30 ± 0.51 ^A,B^	66.19 ± 2.54 ^A,B^	17.58 ± 0.10 ^A^	19.70 ± 0.10 ^B^
Octadecanoic acid C18:0	3.39 ± 0.11 ^A^	26.47 ± 12.14 ^A,B,C^	1.55 ± 0.02 ^B^	2.20 ± 0.08 ^C^
Nonadecenoic acid C19:1	0.03 ± 0.01 ^A^	0.04 ± 0.00 ^B^	ND^A,B,C^	0.02 ± 0.00 ^C^
Nonadecanoic acid C19:0	ND	ND	ND	0.16 ± 0.21
Eicosapentaenoic acid C20:5	1.81 ± 0.27 ^A^	2.84 ± 0.06 ^A^	0.64 ± 0.06 ^A^	1.29 ± 0.12 ^A^
Eicosatetraenoic acid C20:4	5.28 ± 0.06 ^A,B^	12.56 ± 0.33 ^A,B^	1.41 ± 0.15 ^A^	1.65 ± 0.07 ^B^
Eicosatrienoic acid C20:3	0.08 ± 0.00 ^A^	0.05 ± 0.00 ^A^	ND ^A^	0.04 ± 0.00 ^A^
Eicosenoic acid C20:1	0.06 ± 0.01 ^A,C^	0.07 ± 0.01 ^B,D^	ND ^A,B^	0.02 ± 0.00 ^C,D^
Eicosanoic acid C20:0	0.13 ± 0.00 ^A^	0.61 ± 0.09 ^A,B,C^	0.06 ± 0.01 ^B^	0.14 ± 0.00 ^C^
Docosanoic acid C22:0	0.17 ± 0.01 ^A^	0.42 ± 0.01 ^A,B^	0.07 ± 0.00 ^A,B^	0.15 ± 0.00 ^B^
Tetracosenoic acid C24:1	ND ^A^	0.05 ± 0.01 ^A,B^	ND ^B^	0.03 ± 0.00 ^A,B^
Tetracosanoic acid C24:0	0.53 ± 0.02 ^A^	0.63 ± 0.05 ^A^	0.18 ± 0.00 ^A^	0.27 ± 0.00 ^A^
Hexacosanoic acid C26:0	0.38 ± 0.02 ^A^	0.28 ± 0.03 ^A^	0.10 ± 0.01 ^A^	0.19 ± 0.00 ^A^
Octacosanoic acid C28:0	0.35 ± 0.02 ^A^	0.22 ± 0.02 ^A^	0.08 ± 0.00 ^A^	0.15 ± 0.01 ^A^
Triacontanoic acid C30:0	0.29 ± 0.01 ^A^	0.16 ± 0.02 ^A^	0.05 ± 0.00 ^A^	0.10 ± 0.00 ^A^
Dotriacontanoic acid C32:0	0.23 ± 0.02 ^A,B^	ND ^A^	0.02 ± 0.01 ^B^	0.07 ± 0.00 ^A,B^
Sum of FFA	142.42 ± 1.71 ^A,B^	244.96 ± 17.08 ^A,B^	54.81 ± 0.47 ^A^	63.19 ± 0.76 ^B^

FFA—free fatty acid; SD—standard deviation; ND—not detected; trace amount below or equal to 0.004 μg/g of insect body; statistically significant differences (ANOVA, post hoc Tukey’s HSD) between concentration of FFAs are marked with the same letters (^A,B,C,D^); see Supplementary Table S2 for raw data.

**Table 6:** Other compounds extracted from *Sarcophaga argyrostoma* adults (μg/g of insect body ± SD).

	Cuticular		Internal	
	Control	Exposure to *C. coronatus*	Control	Exposure to *C. coronatus*
Glycerol	0.10 ± 0.00 ^A^	0.19 ± 0.01 ^A,B^	0.08 ± 0.01 ^B^	0.07 ± 0.00 ^A^
Cholesterol	6.47 ± 0.08 ^A^	8.50 ± 0.11 ^A^	3.12 ± 0.07 ^A^	3.52 ± 0.03 ^A^

SD—standard deviation; ND—not detected; statistically significant differences (ANOVA, post hoc Tukey’s HSD) between concentration of compounds are marked with the same letters (^A,B^); see Supplementary Table S2 for raw data.

## Data Availability

All data generated or analysed during this study are included in this published article (and its supplementary information files).

## Supplementary Materials

The following are available online at https://www.mdpi.com/article/10.3390/insects12110970/s1. Table S1. Susceptibility of S. argyrostoma to fungal infection—raw data and statistics. Table S2. A comparison of the FFA profiles of the cuticle surface (sum of extracts I and II) and the internal structures of the pupae and imagines—raw data and statistics.
